# Value of repeat renal biopsy in the evaluation of AL amyloidosis patients lacking renal response despite of complete hematologic remission: a case report and literature review

**DOI:** 10.1186/s12882-022-02752-4

**Published:** 2022-03-31

**Authors:** Ping Zhang, Xiuling Chen, Yurong Zou, Wei Wang, Yunlin Feng

**Affiliations:** 1 Nephrology Department, Sichuan Provincial People’s Hospital, Chengdu, 610072 China; 2 Medical School, University of Electronic Science and Technology of China, Chengdu, 611731 China

**Keywords:** AL amyloidosis, Hematologic response, Repeat renal biopsy, Case report, Review

## Abstract

**Backgrounds:**

Published literatures on repeat renal biopsy of AL amyloidosis have basically reached a consensus that amyloid material deposit does not disappear or diminish after satisfactory hematologic response, regardless of renal response. However, the need of a repeat renal biopsy in such situation is still controversial.

**Case presentation:**

Here we reported a case of histologically confirmed λ Type renal AL amyloidosis who had been classified as Stage I and low risk at initial diagnosis. The patient received a total of six courses of CyBorD chemotherapy. She had achieved complete hematologic remission after two courses of chemotherapy but consistently had large amount of proteinuria over 10 g/day during follow up. A repeat renal biopsy was performed nine months after the first one and indicated mild to moderate increase of amyloid deposits as well as significant glomerulosclerosis and interstitial lesions, suggesting a lack of histological renal improvement despite her satisfactory hematologic response.

**Conclusions:**

This case indicated renal involvement in AL amyloidosis could progress after successful hematologic treatment, and supported the value of repeat renal biopsy in the evaluation of AL amyloidosis patients lacking renal response despite of complete hematologic remission.

**Supplementary Information:**

The online version contains supplementary material available at 10.1186/s12882-022-02752-4.

## Background

Renal involvement can be found in two thirds of AL amyloidosis patients [1]. Clinic manifestations range from mild proteinuria to nephrotic syndrome to renal failure [1–3]. Organ response following hematologic response is a key component of successful treatment, and length of survival increases with the depth of organ responses [4]. However, whether treatment for AL amyloidosis can achieve improvement in renal histology is still controversial [5–8].

Lacking of renal response despite of complete hematologic remission is a great treatment challenging. Repeat renal biopsy might be a helpful strategy to investigate such situation. Published literatures on repeat renal biopsy of AL amyloidosis have basically reached a consensus that amyloid material deposit does not disappear or diminish after satisfactory hematologic response with or without renal response [5, 6, 8–10], however, current positions on performing repeat renal biopsy are mixed. In a case series study with the largest sample size so far, Angel-Korman et al. concluded repeat renal biopsy is unnecessary in most cases and would put the patient at risk of bleeding [5]. Other studies, on the other hand, suggested repeat renal biopsy is helpful to evaluate microstructural changes of renal lesions, thus assisting medical decision making [2, 3, 11].

Here we reported a case of AL amyloidosis who consistently had large amount of proteinuria but achieved rapid and complete hematologic remission. Her repeat renal biopsy only nine months after the first one indicated mild to moderate increase of amyloid deposits as well as significant glomerulosclerosis and interstitial lesions, suggesting a lack of histological renal improvement despite her satisfactory hematologic response. Literature review was also performed to summarize published cases of repeat renal biopsy in AL amyloidosis and compared with the present case. Our case provided evidence for progression of renal involvement in AL amyloidosis after achieving complete hematologic remission, and supported the value of repeat renal biopsy in evaluation of AL amyloidosis patients with appropriate indications and low risk of bleeding.

## Case presentation

This patient was a 46 years old lady with a past history of bilateral knee osteoarthritis who presented on February 2021 with a chief compliant of intermittent edema in both lower extremities for nearly one year. She began to notice significant and progressive foamy urine from one month before presentation. The patient was then admitted for further examination. She denied fever, bone pain, fatigue or change in body weight. She also denied chronic use of pain killers or Chinese traditional medicine. Her social history and family history were unremarkable.

**Fig. 1:**
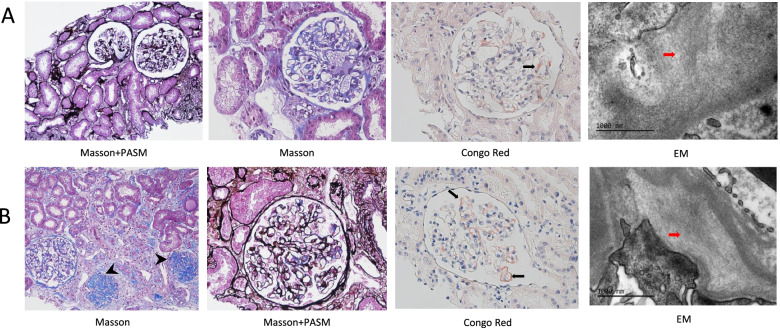
Representative images of renal pathology. **A** The first biopsy: Homogenous amorphous material was noticed in segmental mesangium, blue with Masson stain, and positive for Congo red (black arrow). EM examination showed focal deposit of randomly disposed, non-branching fibrils (9.2–10.0 nm) involving epithelial zones (red arrow). **B** The second biopsy: Beside the same homogenous amorphous material (black arrow) seen in the first biopsy, glomerulosclerosis was observed (black arrow head). EM examination also indicated focal deposit of randomly disposed, non-branching fibrils (10.0 nm) predominately involving epithelial zones (red arrow).

Physical examination at admission indicated a chronically ill-appearing lady in no acute distress, with stable vital signs. She was awake, alert and oriented. Pulmonary, cardiac and abdominal examinations were unremarkable. There was no increase in jugular venous pressure. Moderate pitting edema was noted in both lower extremities. Neurological examination was unremarkable. Results of lab tests at admission including complete blood count (CBC), renal and liver panels, electrolytes, autoimmune-antibodies, and serum complements were all within normal ranges. Serum albumin was 27.1 g/L. 24 h urine protein excretion (24 h UPE) was 11.2 g. Urine albumin-to-creatinine ratio (ACR) was 4818.53 μg/mg. Estimated glomerular filtration rate (eGFR) was 77.5 ml/min/1.73m^2^ by CKD-EPI equation [12]. Serum protein electrophoresis (SPE) found monoclonal (M) proteins in γ region, which was further confirmed as IgA λ type by immunofixation electrophoresis (IFE). Levels of serum free light chain (FLC) for λ and κ chains were 196 and 23.1 mg/L, respectively. Plasma brain natriuretic peptide (BNP) level was normal. Electrocardiogram did not find any abnormality. Echocardiography revealed normal cardiac function, without increase of echo brightness in cardiac walls. Aspirate bone marrow smear found plasma cells accounted for 5%. Flow cytometry analysis of bone marrow indicated 0.49% abnormal plasma cells. The first renal biopsy was performed. Light microscopic examination showed the specimen contained 11 glomeruli with mild mesangial matrix expansion and focal interstitial fibrosis and tubular atrophy (about 5%) (Fig. 1A; for more representative images, see Supplementary Fig. 1). Glomerulosclerosis or arteriolosclerosis was not noticed. Homogenous amorphous material was observed in segmental mesangium, blue with Masson stain, and positive with Congo red. Amyloid deposits on Immunofluorescence (IF) staining were positive with λ type light chain and negative with κ light chain. IF staining was nonspecific for IgG, IgA, IgM, C3, and C1q. Electron microscopy (EM) analysis found focal subepithelial deposit of randomly disposed, non-branching fibrils (9.2–10.0 nm in diameter) and segmental podocyte foot process effacement. The patient was diagnosed λ Type renal AL amyloidosis [13]. Based on 2012 Mayo Prognostic Staging system [14], she was classified as Stage I and low risk, and the predicted median survival would be 94 months.

After thorough discussion with the patient and her families about treatment options including autologous hematopoietic stem cell transplantation (AHSCT) and available chemotherapy regimens, the patient refused AHSCT due to finical reasons and chose chemotherapy. CyBorD regimen consisting of cyclophosphamide 300 mg/m^2^ BSA (body surface area), bortezomib 1.3 mg/m^2^ BSA and dexamethasone 20 mg, was started in March 2021. The treatment was once per week, and four weeks per course. Her contaminant medicine included losartan 75 mg once daily.

**Fig. 2:**
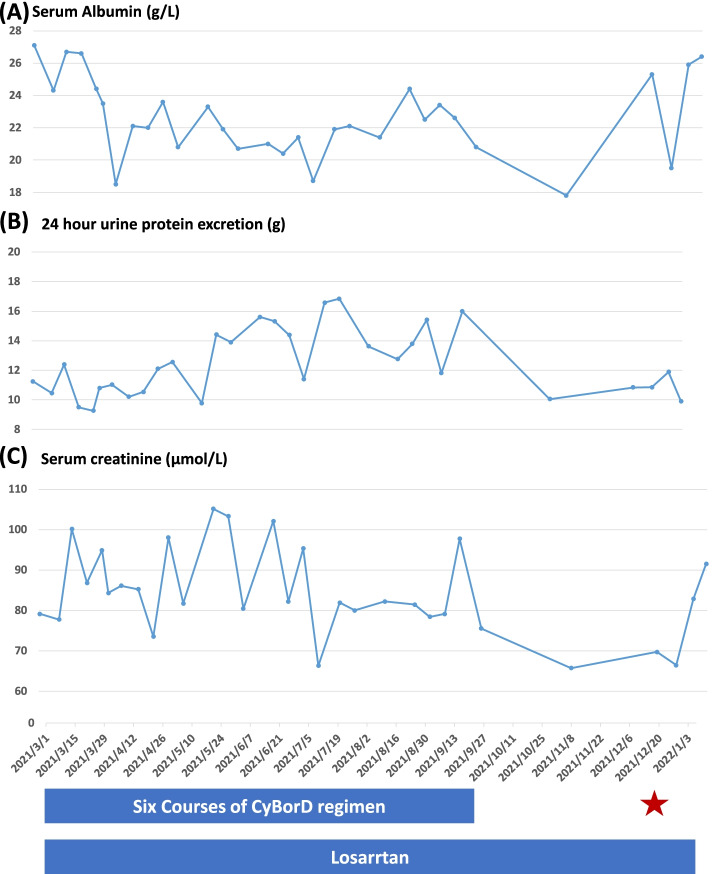
Changes of serum albumin, serum creatinine, and proteinuria over time after the first renal biopsy. Note: The red star indicated the second renal biopsy

The chemotherapy was well tolerated. Any adverse event such as infection or peripheral neuropathy was not observed during the treatment. Changes of important lab variables over time during the follow up duration are shown in Fig. 2 (A-C). At the end of the second course of chemotherapy, her serum κ/λ FLC ratio returned to normal level (0.41) and maintained afterwards. Aspirate bone marrow smear did not identify plasma cell dyscrasia, nor did bone marrow flow cytometry. Complete hematologic response (CR) [15] was achieved. At the end of the third course of chemotherapy, the IFE result became negative and maintained afterwards. Urinary κ/λ FLC ratio was also in the normal range (1.28). Despite the satisfactory efficacy in hematology, her consistently had large amount of proteinuria which kept over 10 g/day. Serum creatinine (Cr) fluctuated within normal range. She still had intermittent mild to moderate edema in both lower extremities. Chemotherapy was stopped after completion of six courses, and the patient continued with her losartan treatment. Three months later, regular follow up indicated her proteinuria was still 10.8 g per day.

To further assess the underlying reasons for such consistently large amount of proteinuria, a second renal biopsy was performed in December, 2021(nine months after the first). Light microscopy examination showed the specimen contained 23 glomeruli, among which 13 were globally sclerotic and obsolescent, and one had fibro-cellular crescent formation (Fig. 1B; for more representative images, see Supplementary Fig. 2). The percentage of focal interstitial fibrosis and tubular atrophy increased from 5% at the first biopsy to 10–15%. Mild and uneven thickening of arteriole wall was noticed. Amyloidosis material deposits were observed in mesangial zones, capillary wall, and interstitial zones. We used a published semi-quantitative scoring system [16] to assess changes of the amyloid load, and found the amyloid score (AS) increased from 1.37 at the first renal biopsy to 3.0 at the second biopsy (for representative images, please see Supplementary Fig. 3), suggesting an mild to moderate increase of amyloid load. Precursor of the amyloidosis was again confirmed as λ type light chain by IF. Focal deposits of randomly distributed, non-branching fibrils (10.0 nm in diameter) were seen under EM, dominantly in subepithelial zones. Taken together, compared with the first biopsy, the second biopsy revealed mild to moderate increase of amyloid material deposits, significant glomerulosclerosis, and mild increase of interstitial fibrosis and tubular atrophy (IFTA). The results of SPE, IFE, aspirate bone marrow smear, and bone marrow flow cytometry during this admission were all negative. Serum levels of λ and κ FLCs were 56 and 41 mg/L, respectively, with a κ/λ ratio of 0.75. AHSCT was again proposed, however, refused by the patient and her families. Chemotherapy was stopped due to the concern about the potentially higher risk over benefit of continuous chemotherapy. The patient continued with losartan which had been titrated to 75 mg twice daily. She is under our close follow up and has been doing well. The latest 24 h urine protein excretion was 9.9 g.

## Discussion and Conclusions

Here we reported a case of repeat renal biopsy of AL amyloidosis who had consistently large amount of proteinuria despite of rapid and complete hematologic response. Renal pathology of the repeat biopsy only nine months after the first one indicated mild to moderate increase of amyloid deposits as well as significant glomerulosclerosis and interstitial lesions, suggesting a lack of histological renal improvement despite her satisfactory hematologic response.

This case has two distinct characteristics compared with published cases in literature (Table 1). First, despite the shortest interval from the first to the second biopsy so far, we found significant progression of glomerulosclerosis (from nearly 0 to 50%) and IFTA (from 5% to 10–15%) suggesting chronicity. The histologic evolution of renal lesions was accompanied by rapid and complete hematologic response. Our findings provide evidence that renal lesions in amyloidosis can progress despite of satisfactory treatment from a hematologic perspective. Meanwhile, the histologic progression might involve not only amyloid deposits, but also other microstructures of nephrons. However, it should be noted sampling differences must be considered while interpreting repeat biopsies, since renal biopsies are performed in renal parenchyma in a rather random manner. Substantial discordance between two biopsies might possibly originate from sampling inconsistency.

**Table 1:** Literature review of repeat renal biopsy for AL amyloidosis and comparison with the present case

Author	Year	No	Treatment	From onset of symptoms to start of chemotherapy	Urinary Protein(g/d)/sCr(mg/dl) at 1^st^ Biopsy	At 2^nd^ Biopsy		From 1^st^ to 2^nd^ Biopsy (year)	Reason for 2^nd^ Biopsy	Changes on Renal Pathology *			
						Hematologic response^#^	Urinary Protein(g/d)/sCr(mg/dl)			Amyloid deposit	Glomerulosclerosis	Arteriolosclerosis	IFTA
Kyle et al. [9]	1982	1	MP	2 y 4 m	3.9/1.98	+	5.1/1.0	6*	NR	↑	NR	NR	NR
		2	MP	8 m	5.0/3.2	+	6.0/1.0	4	NR	↑	NR	NR	NR
Yamazaki et al.[7]	2009	1	VAD + HM/AHSCT	NR	8.0/1.0	+	0.55/1.0	1.3	↓proteinuria	→	NR	NR	NR
Okuyama et al.[6]	2013	1	VRD + HM/AHSCT	NR	3.4/0.6	CR	3.4/0.6	1.4*	NR	→	NR	NR	NR
Nakayama et al.[8]	2005	1	MP	3 m	10/1.3	+	0.7/1.7	3.2	AKI with hemodialysis	↓↓	↑	NR	NR
Roth et al.[17]	2013	1	AHSCT	NR	12.6/1.4	CR	3.0/3.5	9	↑proteinuria, ↑serum Cr	↑↑	↑↑	↑	↑↑
		2	AHSCT	2 m	0.6/2.8	CR	NR/4.7	2	↑serum Cr	↑	↑	↑	↑↑
Zeier et al.[18]	2003	1	Ifosfamide, HM/AHSCT	2 m	7/NR	NR	1.6/NR	3.3	Persistent proteinuria	→	NR	NR	NR
		2	Ifosfamide, HM/AHSCT	1 m	8/2.0	NR	0.6/NR	3.3	Persistent proteinuria	→	NR	NR	NR
Safadi et al. [10]	2015	1	HM/AHSCT	2 m	10/1.8	CR	4/2.1	4	↑serum Cr	→	→	↑**	↓
		2	HM/AHSCT, bortezomib and steroids	1 m	2.5/1.9	CR	3.8/6	1.4	↑serum Cr	→	→	→	↑
Angel-Korman et al. [5]	2020	1	HM/AHSCT	0 y	7.8/1	CR	1.3/3.1	3.0	AKI	↑	→	↑	↑
		2	HM/AHSCT	0 y	15.5/1	CR	4.7/2.4	9.3	↑proteinuria and serum Cr	↑	↑	→	↑
		3	HM/AHSCT	0 y	5.3/0.7	CR	13/0.9	7.7	↑proteinuria	↑	↑	↑	↑
		4	HM/AHSCT	0 y	5.9/1.7	VGPR	7/2.7	3.1	↑serum Cr	↑	↑	↑	↑
		5	HM/AHSCT	0 y	3.3/0.8	VGPR	2/2.5	11.3	↑proteinuria and serum Cr	↑↑	→	→	↑↑
		6	Rituximab, orpozomib, bendamusitine	2.0 y	20/2.9	VGPR	11/4.1	3.5	↑proteinuria and serum Cr	↑	↑	→	→ /↑
		7	Bor-Dex	0 y	13/1.7	CR	1.5/2.5	2.4	↑serum Cr	→	→	→	↑
		8	Len-Dex, HM/AHSCT, Len-Dex, Bor-Dex	0 y	9.7/1.3	VGPR	18/1.2	5.2	↑proteinuria	→	→	→	→
Torui et al.[19]	2020	1	VAD + HM + AHSCT	4 y	4.89/0.9	CR	0.03/0.9	3	To determine the need of further therapy	↓	↑	NR	NR
Present case	2021	1	CyBorD	1 m	11.24/0.89	CR	9.5/1.0	0.75	Persistent high proteinuria	→ /↑	↑↑	↑	↑

*Abbreviations: AHSCT* autologous hemopoietic stem cell transplantation, *AKI* acute kidney injury, *Bor-Dex* bortezomib-dexamethasone, *Cr* creatinine, *CR* complete regression, *CyBorD* cyclosporin, bortezomib, dexamethasone, *F/U* follow up, *HM* high dose melphalan, *IFTA* interstitial fibrosis and tubular atrophy, *Len-dex* lenalidomide-dexamethasone, *m* month(s), *MP* melphalan and prednisone, *NR* not reported, *VAD* vincristine, doxorubicin, and dexamethasone, *VGPR* very good partial regression, *y* year

^*^ ↑indicates increase, ↓ indicates decrease, → indicates virtually the same;**considered as hypertensive arteriosclerosis by original publication; ^#^ For studies before 2012 when hematologic response was clearly defined, + is used to indicate hematologic improvement, including but not limited to decrease/disappearance of serum/urine M proteins, decrease of serum/urine free light chains, and disappearance of plasma cell dycrasia on bone marrow smear. For studies after 2012, hematologic responses are defined based on criteria proposed by[20] et al. and Muchtar et al.[15]

Second, rapid development of prominent glomerulosclerosis and progression of interstitial injury were observed after such short time interval. The driven factor might be the consistently large amount of proteinuria. Secondary FSGS due to amyloid-induced podocyte injury cannot be excluded although typical FSGS manifestations were not observed. Literature review on repeat renal biopsy of AL amyloidosis indicated the majority of published cases had experienced transient or permanent proteinuria reduction before the second biopsy. Among the reported reasons for repeat renal biopsy including this case (Table 1), 33.3% (6/18) was recurrent increase of proteinuria after prior improvement. The amounts of proteinuria in two reported repeat biopsies due to persistent proteinuria were only 0.6 and 1.6 g, much less than that in our case. Roth et al. reported a case with nephrotic syndrome range proteinuria showed significant glomerulosclerosis and IFTA on repeat renal biopsy [17], further supporting that large amount of proteinuria might drive the progression of renal involvement. Another case reported by Torui et al. [19] also revealed progression of glomerulosclerosis, however, significant regression of amyloid deposits in this case was quite a distinct presentation.

The majority of published literature including our case indicated that amyloid deposit did not disappear or regress after hematologic CR [5–10, 17, 18]. Theoretically, amyloid material deposit does not respond to current clone-target chemotherapies. It has been suggested that proteinuria and podocyte pathologies in AL amyloidosis are caused by factors other than amyloid materials per se [21]. Hetzel et al. reported a case of AL amyloidosis that had been diagnosed as minimal change glomerulonephritis two years before confirmation of AL amyloidosis by a second renal biopsy, and suggested mediators such as cytokines might be released during early stage of amyloid deposit formation and cause pathological abnormalities even before renal amyloidosis could be detected [21], parallel to the assumption that altered lymphokine leaded to glomerulonephritis in lymphomas [22, 23]. However, two studies [8, 19] indeed confirmed regression of amyloid deposits. It might be possible to hypothesize that the extent of amyloid deposits does not always correlates to the severity of disease, but more evidence is needed.

Therefore, determining the improvement of renal involvement in AL amyloidosis based on changes of amyloid deposit extent is not an appropriate strategy. This might be the reason that repeat renal biopsy was considered ‘unnecessary in most cases’ by Angel-Korman et al. [5]. However, we had different opinions. A lack of renal response does not necessarily mean an increase in amyloid deposits. The purpose of repeat renal biopsy is not to differentiate between new versus old amyloid deposits, but to find other histologic clues for the lack of renal response. The reason for the lack of renal response despite of hematologic remission might be other microstructure injuries other than amyloid deposits. It has been shown that significant pathology progressions did occur after satisfactory hematologic response with or without contaminant renal response [5, 7, 17]. In our case, the repeat biopsy also detected significant manifestations of glomerulosclerosis and IFTA. It can be difficult to tell whether the lack of renal response is due to progression of existing lesions or an alternative diagnosis, especially when clinic and lab investigations do not provide helpful clues. Therefore, the value of repeat renal biopsy should not be denied due to the failure to find regression of amyloid deposit. In the contrary, repeat renal biopsy could not only assess progression or severity of existing renal involvement, but also detect potentially new lesions. In addition, with the guidance of ultrasound, renal biopsy is relatively safe in patients without high risk of bleeding [24, 25]. Pathologic results can help us carefully balance between the benefits and risks of continuing chemotherapy, thus protect patients from unnecessary adverse effects. Interestingly, there were two studies reporting resolution of amyloid deposit after treatment of melphalan and prednisone [8, 19]. However, both studies also reported markedly increase of glomerulosclerosis and claimed resolution of amyloid deposit could not explain the progression of the disease.

Achievement of hematologic response, including complete or very good partial remission, is a requirement for organ response [4]. The reported median time to renal response in AL amyloidosis was six months [4], albeit often in a delayed manner since current therapies target plasma cells instead of amyloid deposits in affected organs. The best treatment duration for patients who have achieved hematologic complete response but not renal response is inconclusive yet. In China, AHSCT is suggested as soon as hematologic response has been reached, to acquire maximum response [1]. For patients who are not AHSCT candidates and have reached complete hematologic response, the suggested regimen is six courses of chemotherapy and then waiting for organ response. However, the appropriate length of this ‘wait and see’ strategy is unclear yet. There is also a lack of variables that can help us predict whether organ response will be achieved. In this study, based on the pathologic results from repeat renal biopsy that indicated rapid and significant progression of renal lesions, although the patient has been doing well and was classified as prognosis Stage I and low risk, we suspect the patient might develop renal insufficiency in the not too distant future. The importance of organ response on overall survival of AL amyloidosis patients have also been reported [4]. The patient reported herein should be classified as non-responder based Muchtar et al.’s criteria [4].

In summary, we reported a case of AL amyloidosis whose repeat renal biopsy only nine months after the first one indicated mild to moderate increase of amyloid deposits and significant glomerulosclerosis and interstitial lesions, suggesting a lack of histological renal improvement despite her complete hematologic response. This case indicated renal involvement in AL amyloidosis could progress after successful hematologic treatment, and supported the value of repeat renal biopsy in the evaluation of AL amyloidosis patients lacking renal response despite of complete hematologic remission.

## Supplementary Information

**Additional file 1.** Representative images of the first renal biopsy. Light microscopy found mild mesangial matrix expansion and focal interstitial fibrosis and tubular atrophy (about 5%). No glomerulosclerosis or arteriolosclerosis was noticed. Homogenous amorphous material was noticed in segmental mesangium, blue with Masson stain, and positive for Congo red. Amyloid deposits on IF were for λ type light chain. The corresponding cross section was negative with κ light chain. EM examination showed focal deposits of randomly disposed, non-branching fibrils (9.2-10.0 nm) involving epithelial zones and segmental podocyte foot process effacement.

**Additional file 2.** Representative images of the second renal biopsy. Among the 23 glomeruli under light microscopy, 13 were globally sclerotic and obsolescent, and one had cellular fibrous crescent formation. Lesions of focal interstitial fibrosis and tubular atrophy increased from 5% at the first biopsy to 10-15%. Thickening of arteriole wall was noticed. Amyloidosis material deposit still existed in mesangium, without any sign of regression. Precursor of the amyloidosis was again confirmed as λ type light chain by IF. Focal deposit of randomly distributed, non-branching fibrils (10.0 nm) predominately involving epithelial zones was seen under EM. Taken together, compared with the results in the first biopsy, there were mild to moderate increase of amyloid material deposit, significant glomerulosclerosis, and mild increase of interstitial fibrosis and tubular atrophy.

**Additional file 3.** Representative images of the semi-quantitative amyloid load assessment. Amyloid deposits in mesangial zones (red arrow), capillary walls (yellow arrow), and interstitial zones (white arrow) and the resulting scores were shown for (A) the first renal biopsy and (B) the second renal biopsy. The scoring tool for amyloid load assessment reported by Rubinstein et al[16] was used to perform this semi-quantitative evaluation. Briefly, the amyloid score (AS) is defined as the sum of scores from four domains, including mesangial (M), capillary wall (CW), interstitial (I) and vascular (V) scores, each of which was scored on a semi-quantitative scale ranging from 0 to 3. The score was based on the percentage of corresponding areas (i.e. M, CW, I, and V) that had been filled with amyloid deposits (0=absent, 1=<25%, 2=25–50%, 3=>50%). Average values were calculated for all non-sclerotic glomeruli, interstitial areas and vessels present and used as the result for each domain.

## Data Availability

The data and materials reported in this work are available on reasonable request to the correspondence.

## Abbreviations

ACR: Albumin-to-creatinine ratio
AHSCT: Autologous hematopoietic stem cell transplantation
BNP: Brain natriuretic peptide
BSA: Body surface area
CBC: Complete blood count
Cr: Creatinine
CR: Complete response
EM: Electron microscopy
FLC: Free light chain
IF: Immunofluorescence
IFE: Immunofixation electrophoresis
IFTA: Interstitial fibrosis and tubular atrophy
M proteins: Monoclonal proteins
SPE: Serum protein electrophoresis
UPE: Urine protein excretion
