# Supplementary material for: Value of repeat renal biopsy in the evaluation of AL amyloidosis patients lacking renal response despite of complete hematologic remission: a case report and literature review
Source: BMC Nephrol. 2022 Mar 31;23:127. doi: 10.1186/s12882-022-02752-4 (PMC8974030; doi:10.1186/s12882-022-02752-4)
Supplement: Supplementary file 1 — Additional file 1. Representative images of the first renal biopsy. Light microscopy found mild mesangial matrix expansion and focal interstitial fibrosis and tubular atrophy (about 5%). No glomerulosclerosis or arteriolosclerosis was noticed. Homogenous amorphous material was noticed in segmental mesangium, blue with Masson stain, and positive for Congo red. Amyloid deposits on IF were for λ type light chain. The corresponding cross section was negative with κ light chain. EM examination showed focal deposits of randomly disposed, non-branching fibrils (9.2-10.0 nm) involving epithelial zones and segmental podocyte foot process effacement. [file 12882_2022_2752_MOESM1_ESM.pdf]

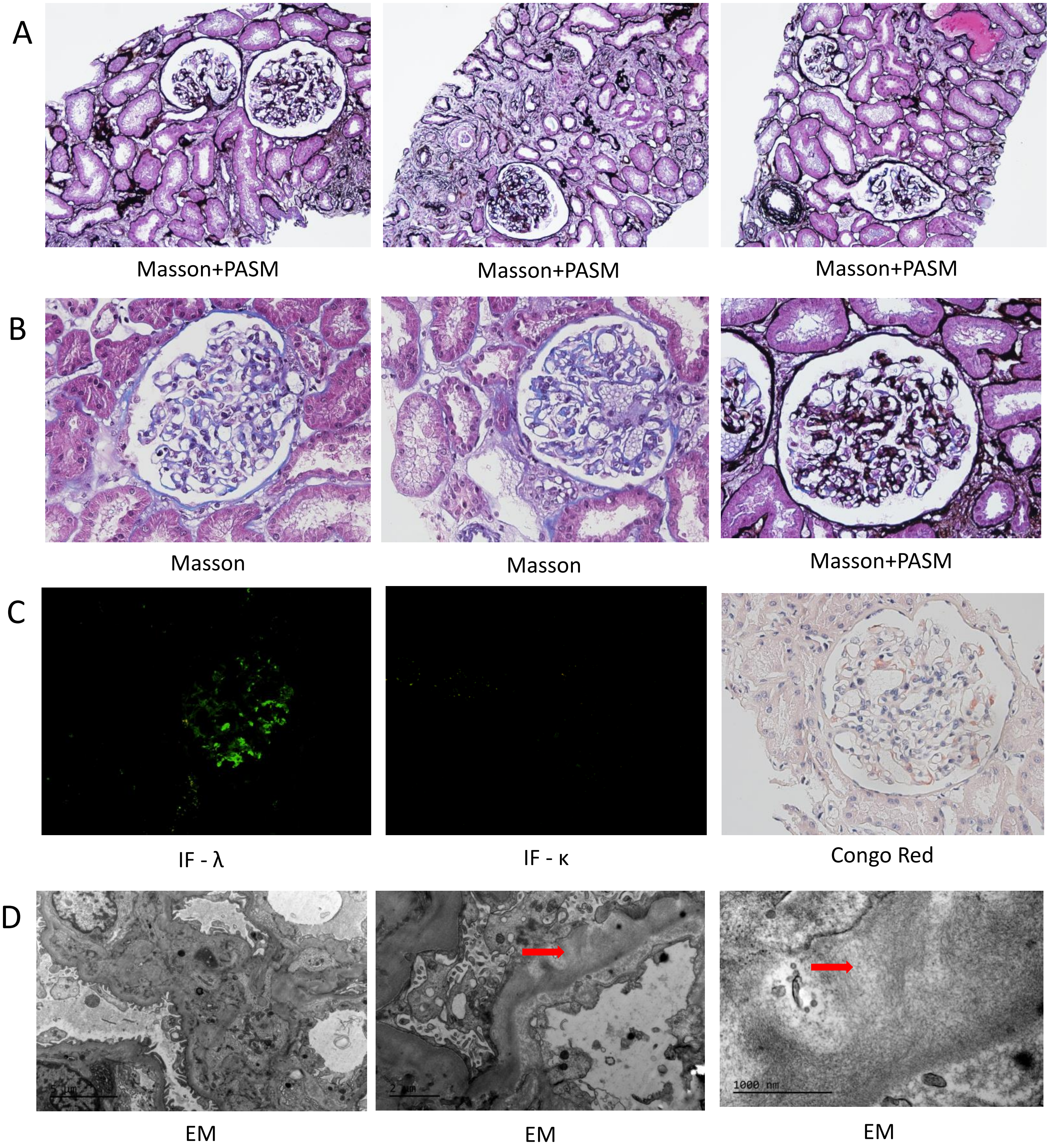
A
Masson+PASM
Masson+PASM
Masson+PASM
B
Masson
Masson
Masson+PASM
C
IF - λ
IF - κ
Congo Red
D
EM
EM
1000 nm
EM
