# Supplementary material for: Value of repeat renal biopsy in the evaluation of AL amyloidosis patients lacking renal response despite of complete hematologic remission: a case report and literature review
Source: BMC Nephrol. 2022 Mar 31;23:127. doi: 10.1186/s12882-022-02752-4 (PMC8974030; doi:10.1186/s12882-022-02752-4)
Supplement: Supplementary file 2 — Additional file 2. Representative images of the second renal biopsy. Among the 23 glomeruli under light microscopy, 13 were globally sclerotic and obsolescent, and one had cellular fibrous crescent formation. Lesions of focal interstitial fibrosis and tubular atrophy increased from 5% at the first biopsy to 10-15%. Thickening of arteriole wall was noticed. Amyloidosis material deposit still existed in mesangium, without any sign of regression. Precursor of the amyloidosis was again confirmed as λ type light chain by IF. Focal deposit of randomly distributed, non-branching fibrils (10.0 nm) predominately involving epithelial zones was seen under EM. Taken together, compared with the results in the first biopsy, there were mild to moderate increase of amyloid material deposit, significant glomerulosclerosis, and mild increase of interstitial fibrosis and tubular atrophy. [file 12882_2022_2752_MOESM2_ESM.pdf]

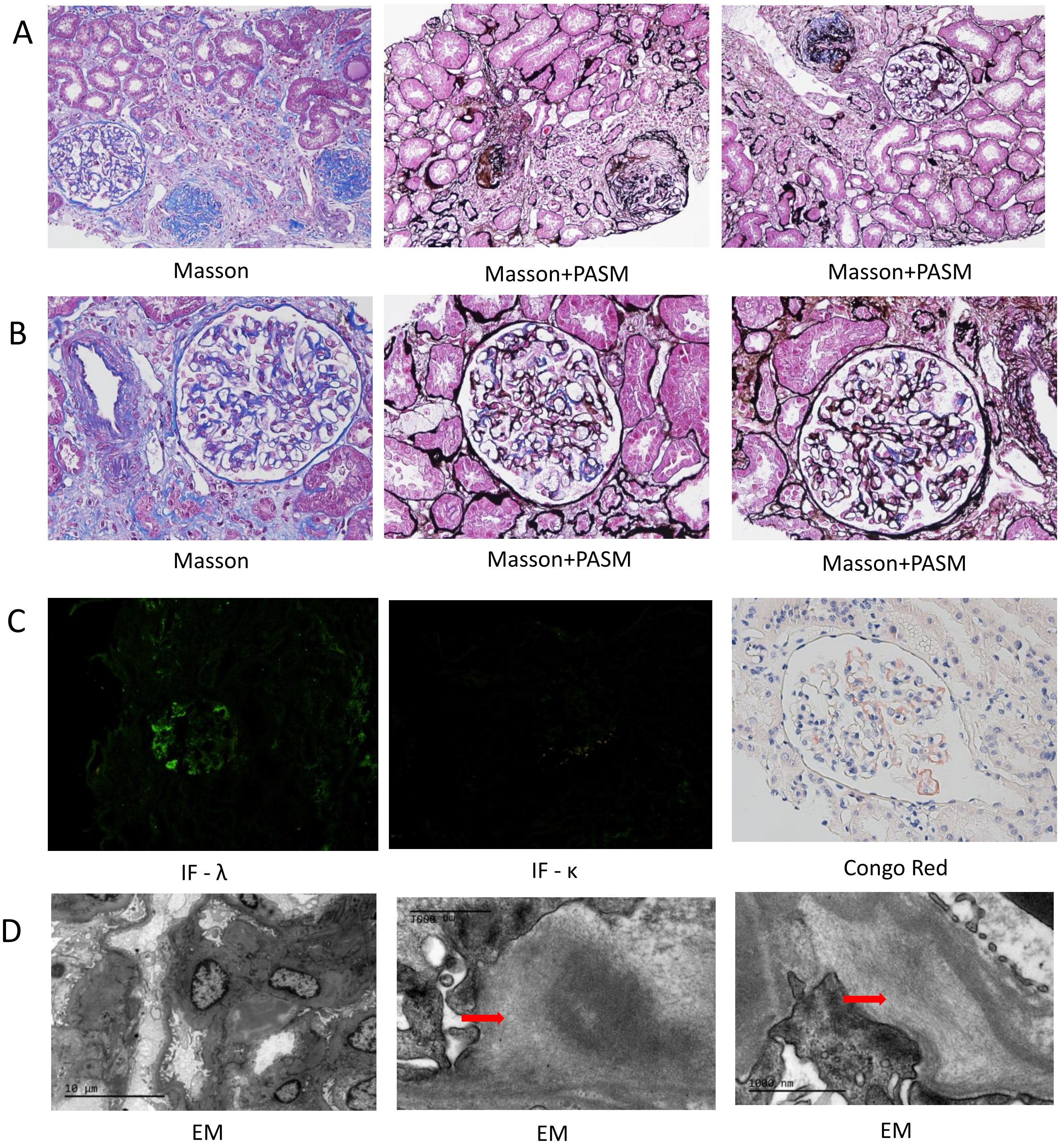
A
Masson
Masson+PASM
Masson+PASM
B
Masson
Masson+PASM
Masson+PASM
C
IF - λ
IF - κ
Congo Red
D
10 μm
1000 nm
1000 nm
EM
EM
EM
