# Supplementary figures and images for: Value of repeat renal biopsy in the evaluation of AL amyloidosis patients lacking renal response despite of complete hematologic remission: a case report and literature review
Source: BMC Nephrol. 2022 Mar 31;23:127. doi: 10.1186/s12882-022-02752-4 (PMC8974030; doi:10.1186/s12882-022-02752-4)

A

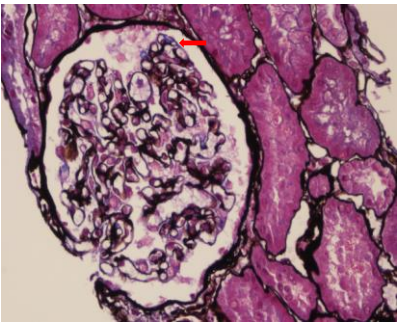

M=1, CW=0

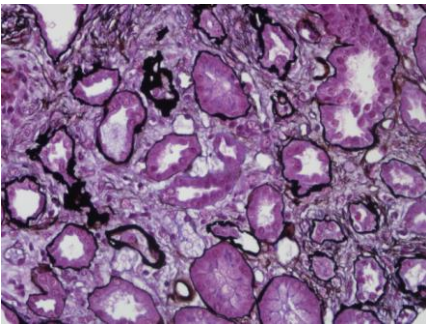

I=0

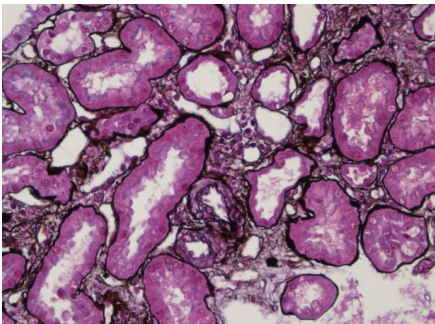

V=0

B

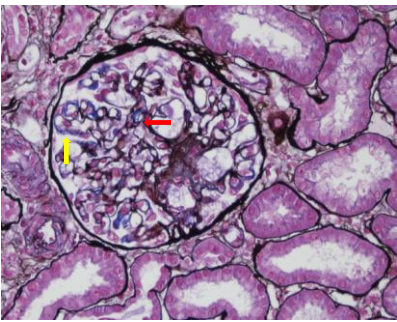

M=1, CW=1

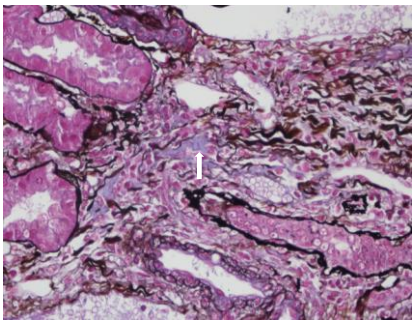

I=1

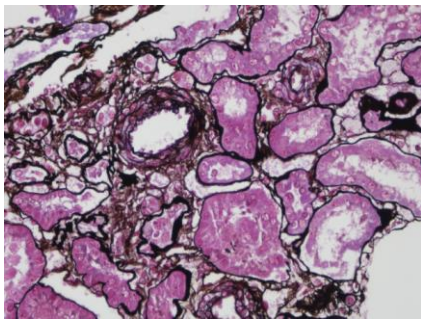

V=0

Supplement: Supplementary file 3 — Additional file 3. Representative images of the semi-quantitative amyloid load assessment. Amyloid deposits in mesangial zones (red arrow), capillary walls (yellow arrow), and interstitial zones (white arrow) and the resulting scores were shown for (A) the first renal biopsy and (B) the second renal biopsy. The scoring tool for amyloid load assessment reported by Rubinstein et al[16] was used to perform this semi-quantitative evaluation. Briefly, the amyloid score (AS) is defined as the sum of scores from four domains, including mesangial (M), capillary wall (CW), interstitial (I) and vascular (V) scores, each of which was scored on a semi-quantitative scale ranging from 0 to 3. The score was based on the percentage of corresponding areas (i.e. M, CW, I, and V) that had been filled with amyloid deposits (0=absent, 1=<25%, 2=25–50%, 3=>50%). Average values were calculated for all non-sclerotic glomeruli, interstitial areas and vessels present and used as the result for each domain. [file 12882_2022_2752_MOESM3_ESM.pdf]
